# Near wall Prandtl number effects on velocity gradient invariants and flow topologies in turbulent Rayleigh–Bénard convection

**DOI:** 10.1038/s41598-020-71665-9

**Published:** 2020-09-10

**Authors:** Sahin Yigit, Josef Hasslberger, Markus Klein, Nilanjan Chakraborty

**Affiliations:** 1 Institute of Applied Mathematics and Scientific Computing, Bundeswehr University Munich, Werner-Heisenberg-Weg 39, 85577 Neubiberg, Germany; 2 School of Engineering, Newcastle University, Claremont Road, Newcastle Upon Tyne, NE1 7RU UK

**Keywords:** Mechanical engineering, Fluid dynamics

## Abstract

The statistical behaviours of the invariants of the velocity gradient tensor and flow topologies for Rayleigh–Bénard convection of Newtonian fluids in cubic enclosures have been analysed using Direct Numerical Simulations (DNS) for a range of different values of Rayleigh (i.e. $Ra=10^7-10^9$) and Prandtl (i.e. $Pr=1$ and 320) numbers. The behaviours of second and third invariants of the velocity gradient tensor suggest that the bulk region of the flow at the core of the domain is vorticity-dominated whereas the regions in the vicinity of cold and hot walls, in particular in the boundary layers, are found to be strain rate-dominated and this behaviour has been found to be independent of the choice of *Ra* and *Pr* values within the range considered here. Accordingly, it has been found that the focal topologies S1 and S4 remain predominant in the bulk region of the flow and the volume fraction of nodal topologies increases in the vicinity of the active hot and cold walls for all cases considered here. However, remarkable differences in the behaviours of the joint probability density functions (PDFs) between second and third invariants of the velocity gradient tensor (i.e. *Q* and *R*) have been found in response to the variations of *Pr*. The classical teardrop shape of the joint PDF between *Q* and *R* has been observed away from active walls for all values of *Pr*, but this behavior changes close to the heated and cooled walls for high values of *Pr* (e.g. $Pr=320$) where the joint PDF exhibits a shape mirrored at the vertical *Q*-axis. It has been demonstrated that the junctions at the edges of convection cells are responsible for this behaviour for $Pr=320$, which also increases the probability of finding S3 topologies with large negative magnitudes of *Q* and *R*. By contrast, this behaviour is not observed in the $Pr=1$ case and these differences between flow topology distributions in Rayleigh–Bénard convection in response to *Pr* suggest that the modelling strategy for turbulent natural convection of gaseous fluids may not be equally well suited for simulations of turbulent natural convection of liquids with high values of *Pr*.

## Introduction

Rayleigh–Bénard configuration is one of the most well-known natural convection problems in enclosed spaces where buoyancy-driven fluid motion takes place between differentially heated horizontal walls with the heated bottom wall. This configuration has been widely analysed because of its conceptual simplicity and relevance to several applications ranging from astrophysics, geophysics and meteorology to process industries. Interested readers can be referred to Bodenschatz et al.^1^ for an extensive review in Rayleigh–Bénard convection. Recently, immense heat transport enhancement (e.g. 500 %) was reported for Rayleigh–Bénard convection applications, by using water-heavy liquid (hydrofluoroether) mixture^2^ and vibration-induced boundary-layer destabilization^3^.

The flow becomes turbulent for high values of Rayleigh number $Ra=\rho ^2c_{p}g\beta \Delta TL^3/\mu k$ where $\rho ,c_{p},\beta ,\mu$ and *k* are density, specific heat, volume expansion coefficient, viscosity and thermal conductivity, respectively and *g*, $\Delta T$ and *L* are the acceleration due to gravity, temperature difference between hot and cold walls and the enclosure height, respectively in the Rayleigh–Bénard convection. In the aforementioned applications, turbulent Rayleigh–Bénard convection is obtained for fluids with different Prandtl numbers $Pr=\mu \,c_{p}/\,k$ (e.g. $Pr\approx 1$ is relevant to weather predictions, whereas $Pr\gg 1$ is relevant to geophysics and process industries).

The relative thicknesses of hydrodynamic and thermal boundary layers is dependent on *Pr*, which is known to affect the scalar spectrum and it is possible to obtain roll-off of the scalar spectrum in the inertial range and an inertial-diffusive range is obtained for $Pr<1$^4^. By contrast, the roll-off of the scalar spectrum is obtained for the length scales smaller than the Kolmogorov length scale and a viscous-diffusive range is observed for the scalar spectrum for $Pr\gg 1$^4^. As the temperature distribution in turbulent flows is affected by *Pr*, it can be expected that the velocity distribution in natural convection will also be affected by *Pr* because the flow is induced by the temperature difference.

The Prandtl number has indeed been shown to affect the turbulent kinetic energy spectrum in a recent analysis by the present authors^5^. However, the Prandtl number of the fluid does not only affect the distribution of turbulent kinetic energy in Rayleigh–Bénard convection but also has the potential to alter the distribution of flow topologies, as they can be categorised in terms of the invariants of the velocity gradient $\partial {u_i}/\partial {x_j}$ tensor (i.e. *P*, *Q* and *R*) where $u_i$ is the *i*th component of the velocity vector^6,7^. Depending on the values of the invariants of the velocity gradient $\partial \ u_i/\partial \ x_j$ tensor, 8 different topologies (i.e. S1–S8 topologies) can be identified in the three-dimensional *P*, *Q*, *R* phase space. The velocity-gradient tensor can be split into symmetric and skew-symmetric parts: $A_{ij}=\partial u_i/\partial x_j=S_{ij}+W_{ij}$, where $S_{ij}=0.5(A_{ij}+A_{ji})$ and $W_{ij}=0.5\left( A_{ij}-A_{ji}\right)$ are the symmetric and skew-symmetric components, which are referred to as strain and rotation rates, respectively. Three eigenvalues, $\lambda _1$, $\lambda _2$ and $\lambda _3$, of $A_{ij}$ can be obtained from solutions of the characteristic equation $\lambda ^3+P\lambda ^2+Q\lambda +R=0$ where *P*, *Q*, *R* are the invariants of $A_{ij}$^6,7^:

1 $$\begin{aligned} P=-\left( \lambda _1+\lambda _2+\lambda _3\right) ; \,Q=0.5\left( (P^2-S_{ij}S_{ij})+{(W}_{ij}W_{ij})\right) =Q_s+Q_w; \,R=(-P^3+3PQ-S_{ij}S_{jk}S_{ki}-3W_{ij}W_{jk}S_{ki})/3 \end{aligned}$$

**Figure 1:**
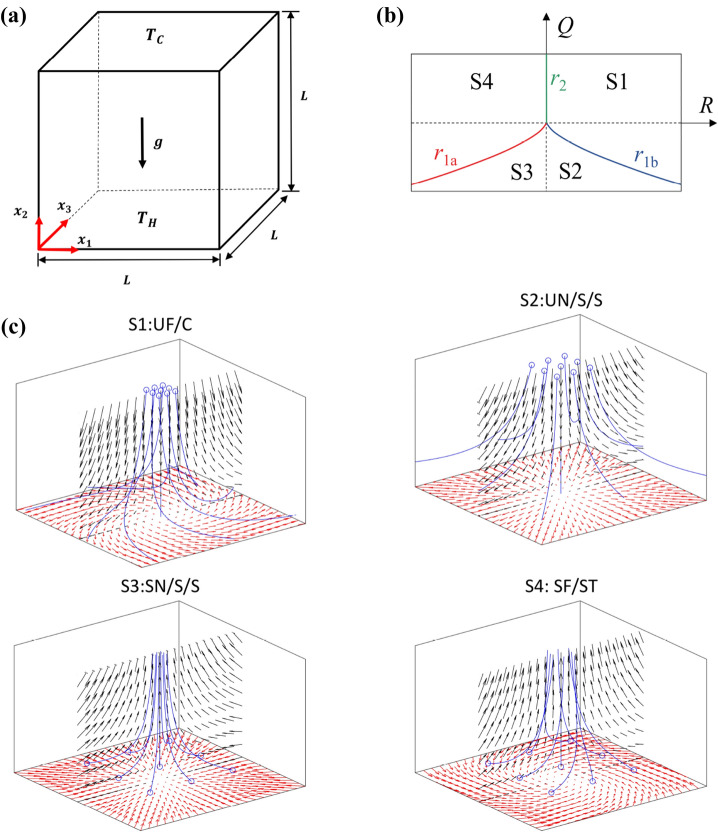
**(a)** Schematic diagram of the simulation domain. **(b)** Classification of topologies S1–S4. **(c)** Graphical representation of topologies S1–S4. Symbols correspond to *UF* unstable focus; *UN* unstable node; *SN* stable node; *SF* stable focus; *C* compressing; *S* saddle; *ST* stretching. The blue circles indicate the origin of the blue streamlines.

The discriminant, $D=\left[ 27R^2+\left( 4P^3-18PQ\right) R+4Q^3-P^2Q^2\right] /108$, of the characteristic equation divides the $P-Q-R$ phase-space into two regions depending on the sign of the discriminant. For $D>0\, (D<0)$, a focal (nodal) topology is obtained^6,7^ and the velocity gradient tensor exhibits one real eigenvalue and two complex conjugate eigenvalues for focal topologies, whereas three real eigenvalues are obtained for nodal topologies. The solutions of $D=0$ are given by two surfaces in the $P-Q-R$ phase space^6,7^: $\ r_{1a}=P\left( Q-2P^2/9\right) /3-2\left( -3Q+P^2\right) ^{{3}/{2}}/27$ and $r_{1b}=P\left( Q-2P^2/9\right) /3+2\left( -3Q+P^2\right) ^{{3}/{2}}/27$. For a positive discriminant (i.e. $D>0$), the $A_{ij}$ tensor has purely imaginary eigenvalues on the surface $r_2$, which is given by $R=PQ$. The surfaces $r_{1a}$, $r_{1b}$ and $r_2$, divide the $P-Q-R$ phase space into eight flow topologies. The first invariant $P=-\partial u_{i}/\partial x_{i}$ of the velocity gradient tensor vanishes for incompressible fluids, and therefore only topologies S1–S4 are observed for $P=0$, as shown in Fig. 1. Therefore, in Rayleigh–Bénard convection of incompressible fluids the flow topologies are determined by the behaviours of the second and third invariants (i.e. *Q* and *R*) of the velocity gradient tensor^6,7^ and only S1–S4 topologies can be seen.

The flow structures associated with S1–S4 topologies are schematically shown in Fig. 1c. One aspect of this work focuses on flow topologies close to the active walls and therefore only the upper part (above the horizontal plane crossing the origin of the coordinate system) of the velocity field is shown in Fig. 1, representative of a situation close to the lower wall, but in principle it can be mirrored at the horizontal plane (see e.g. Ref.^8^). It has been demonstrated by Perry and Chong^6^ and Soria et al.^9^ that S4 topologies are obtained predominantly for positive values of *Q*, whereas Blackburn et al.^10^ demonstrated that the topologies S2 and S4 are predominantly obtained in the regions away from the wall in boundary layer flows. The ‘teardrop’ structure in the joint probability density function (PDF) between *Q* and *R* has been demonstrated by Chong et al.^7^ and Chacin and Cantwell^11^. The analysis by Ooi et al.^12^ and experimental evidences^11,13^ suggested that the same qualitative behaviour is observed in a range of different incompressible turbulent flows indicating some degree of universality in the joint PDFs between *Q* and *R*. The theoretical justifications of the ‘teardrop’ shape of the $Q-R$ joint PDF for incompressible flows have been provided by Elsinga and Marusic^14^ and the loss of ‘teardrop’ structure was shown to be a mark of intermittency in some previous analyses^15^. Tsinober^16^ postulated that the enstrophy production is large in S4 topology whereas the strain rate production is concentrated in regions of S1 topology. The flow topology distributions in Rayleigh–Bénard convection, where temperature and velocity fields are intrinsically coupled, are yet to be analysed in detail^17–20^ in comparison to the vast body of literature (e.g. Refs.^5–16^) on other wall-bounded flows.

The analyses by Dabbagh et al.^17–19^ revealed the existence of the teardrop shape in the bulk region away from the walls in Rayleigh–Bénard convection but the small scale structures in the vicinity of the hot and cold walls have not been discussed there in terms of *Q* and *R*. Xi et al.^20^ reported a transition of flow topologies from a quadruple structure to a dipole structure based on Rayleigh number in turbulent Rayleigh–Bénard convection, which has implications on the Nusselt number (or heat transfer rate). A recent analysis revealed that large-scale circulation in Rayleigh–Bénard convection is affected by Prandtl number^21^. However, the effects of Prandtl number on the flow topology are yet to be analysed and the present work addresses this gap in the existing literature. In this respect, the main objectives of the present analysis are: (a) to demonstrate and explain the effects of Prandtl number on the statistical behaviours of *Q* and *R* and their joint PDFs and (b) to indicate the implications of the above findings on flow topology distribution for Rayleigh–Bénard convection of Newtonian fluids. According to Buckingham’s pi theorem^22^, the Nusselt number $Nu={h\,L}/{k}$, where *h* represents the convective heat transfer coefficient, can be taken to be a function of *Ra* and *Pr* (i.e. $Nu=f(Ra,Pr)$) for Rayleigh–Bénard convection in a cubic enclosure. Therefore, three-dimensional Direct Numerical Simulations (DNS) of Rayleigh–Bénard convection in a cubic enclosure for different values of *Ra* (i.e. $Ra={10}^7-{10}^9$ ) and Prandtl number ($Pr=1$ and 320) have been carried out in order to meet these objectives. It is worth noting that $Pr=320$ corresponds for example to silicone oil at ${20}^{o}C$ which exhibits Newtonian rheological behaviour^23^.

The conservation equations for mass, momentum and energy for incompressible Newtonian fluids under transient conditions take the following form:

2 $$\begin{aligned}&\partial u_{i}/\partial x_{i}=0; \end{aligned}$$

3 $$\begin{aligned}&{[}\partial u_{i}/\partial t + u_{j} \, (\partial u_{i}/\partial x_{j})]= -1/\rho \, (\partial P/\partial x_{i}) + \nu \, [\partial ^2 u_{i}/(\partial x_{j}\,\partial x_{j})] + g[\beta (T-T_{ref})]\, \delta _{i2} ; \end{aligned}$$

4 $$\begin{aligned}&{[}\partial T/\partial t + u_{j} \, (\partial T/\partial x_{j})]= \alpha \, [\partial ^2 T/(\partial x_{j}\,\partial x_{j})]. \end{aligned}$$

The last term on the right-hand side of Eq. (3) originates due to Boussinesq’s approximation and the temperature difference between horizontal walls is considered to be small enough so that this approximation remains valid. Also, in Eq. (3), $\nu$ is the kinematic viscosity and the Kronecker delta ($\delta _{i2}$) indicates that buoyancy forces affect the flow only in the vertical direction (i.e. $x_{2}$ direction). The reference temperature ($T_{ref}$) is taken to be the cold wall temperature (i.e. $T_{ref}=T_{C}$). In Eq. (4), α=k/(ρ cp) is the thermal diffusivity of the fluid.

Equations (2–4) are solved in a coupled manner in conjunction with the following boundary conditions. The simulation configuration is schematically shown in Fig. 1a which demonstrates that the differentially heated horizontal walls are subjected to constant wall temperature boundary conditions (i.e. $T=T_{H}$ at $x_{2}=0$ and $T=T_{C}$ at $x_{2}=L$ where $T_{H}>T_{C}$). All the other walls are considered to be adiabatic (i.e. $\partial T/\partial x_{1,3}=0$ at $x_{1,3}=0,\,L$). Finally, no-slip and impermeability conditions are specified for all walls (i.e. $u_{1,2,3}=0$ at $x_{1,2,3}=0,\,L$).

**Table 1:** Summary of the mean Nusselt number (i.e. ${\overline{Nu}}$) for different values of *Ra* and *Pr* of Rayleigh–Bénard convection of Newtonian fluids in a cubic enclosure.

*Pr*/*Ra*	$10^7$	$10^8$	$10^9$
1	16.39	31.61	62.56
320	17.43	32.80	64.33

## Results

**Figure 2:**
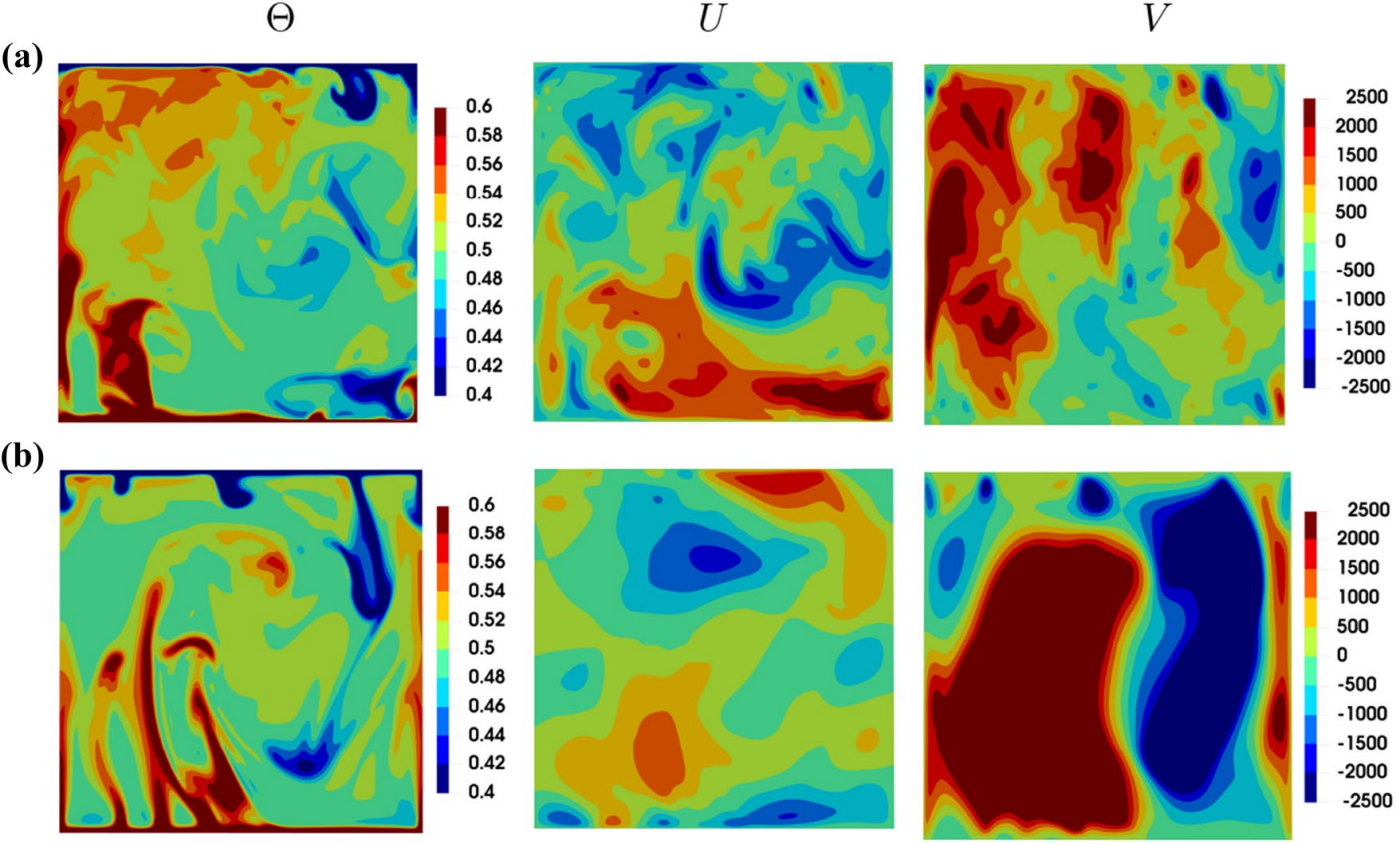
Instantaneous distributions of non-dimensional temperature $\theta$ (left column), non-dimensional horizontal velocity component *U* (middle column) and non-dimensional vertical velocity component *V* (right column) in a $x_1-x_2$ plane for **(a)** $Pr=1$ (top row), **(b)** $Pr=320$ (bottom row) at $Ra={10}^8$.

The instantaneous distributions of non-dimensional temperature $\theta =(T-T_C)/(T_H-T_C)$, non-dimensional horizontal velocity component $U=u_1L/\alpha$ and non-dimensional vertical velocity component $V=u_2L/\alpha$ in the $x_1-x_2$ midplane are exemplarily shown in Fig. 2 for $Ra={10}^8$ in the case of $Pr=1$ and 320. It can be seen from Fig. 2 that strong thermal gradients exist only within the thermal boundary layer close to the active walls and the temperature within the interior of the enclosure remains mostly uniform and close to $\theta =0.5$. This behaviour is indicative of strong turbulent heat transfer in the bulk region of the domain and contrasts with a stratification with isotherms parallel to horizontal walls in the case of pure conduction (not shown here). Moreover, thermal plumes from the active hot and cold walls can be discerned in the $Pr=320$ case, whereas this tendency is less prevalent for the $Pr=1$ case where a large-scale circulation is evident. The strong convective transport within the cavity can be substantiated from the distributions of *U* and *V* in Fig. 2, which reveal that the lighter hot fluid in the vicinity of the hot bottom wall rises in the vertical direction and moves in the horizontal direction while transferring the heat to the cold wall and eventually sinks in the downward direction because of its higher density in comparison to the fluid underneath in the vicinity of the hot wall. Furthermore, Fig. 2 shows that for $Pr=320$ the flow is mushroom-shaped plume-dominated with large scale vertical velocity structures, whereas more mixed (i.e. stronger turbulent) fluid flow prevails with a superimposed large scale circulation in the distributions of *U* and *V* for $Pr=1$. The turbulent flow strengthens with decreasing value of *Pr* for given value of *Ra* in the Rayleigh–Bénard convection^24^. This actually can be explained by the increasing Grashof number (*Gr*, dimensionless number which indicates the ratio of buoyancy to viscous forces acting on a fluid) with decreasing *Pr* values for a given value of *Ra* (i.e. $Gr=Ra/Pr$). Thus, higher values of *Gr* signify the relative augmentation of the buoyancy forces in the fluid domain.

The distributions of temperature and fluid velocities remain qualitatively similar for different values of *Ra* for a given value of *Pr*. However, the magnitude of fluid velocity increases with increasing *Ra*. Figure 2 indicates that distributions of temperature are affected by *Pr*, which is also reflected in a moderate influence of *Pr* on the mean Nusselt number (i.e. ${\overline{Nu}}$), which can be substantiated from Table 1.

The mean Nusselt number is defined as a dimensionless heat flux averaged over horizontal walls and over time as follows:

5 $$\begin{aligned}&h = |{-k \,(\partial T / \partial x_{2})_{wf} }\,/\,{(T_{wall} - T_{ref})}| \, , {\overline{Nu}}=({\langle {h}\rangle _{(A,t)} \,L})/\,{k} , \end{aligned}$$

where subscripts ‘wf’ refers to the condition of the fluid in contact with the wall, $T_{wall}$ is the wall temperature and $T_{ref}$ is the appropriate reference temperature, which can be taken to be the temperature of the other wall. In Eq. (5), the mean value of the Nusselt number on the walls (i.e. ${\overline{Nu}}$) is obtained by averaging convective heat transfer coefficients ($\langle {h}\rangle _{(A,t)}$) over horizontal active walls and over time here.

**Figure 3:**
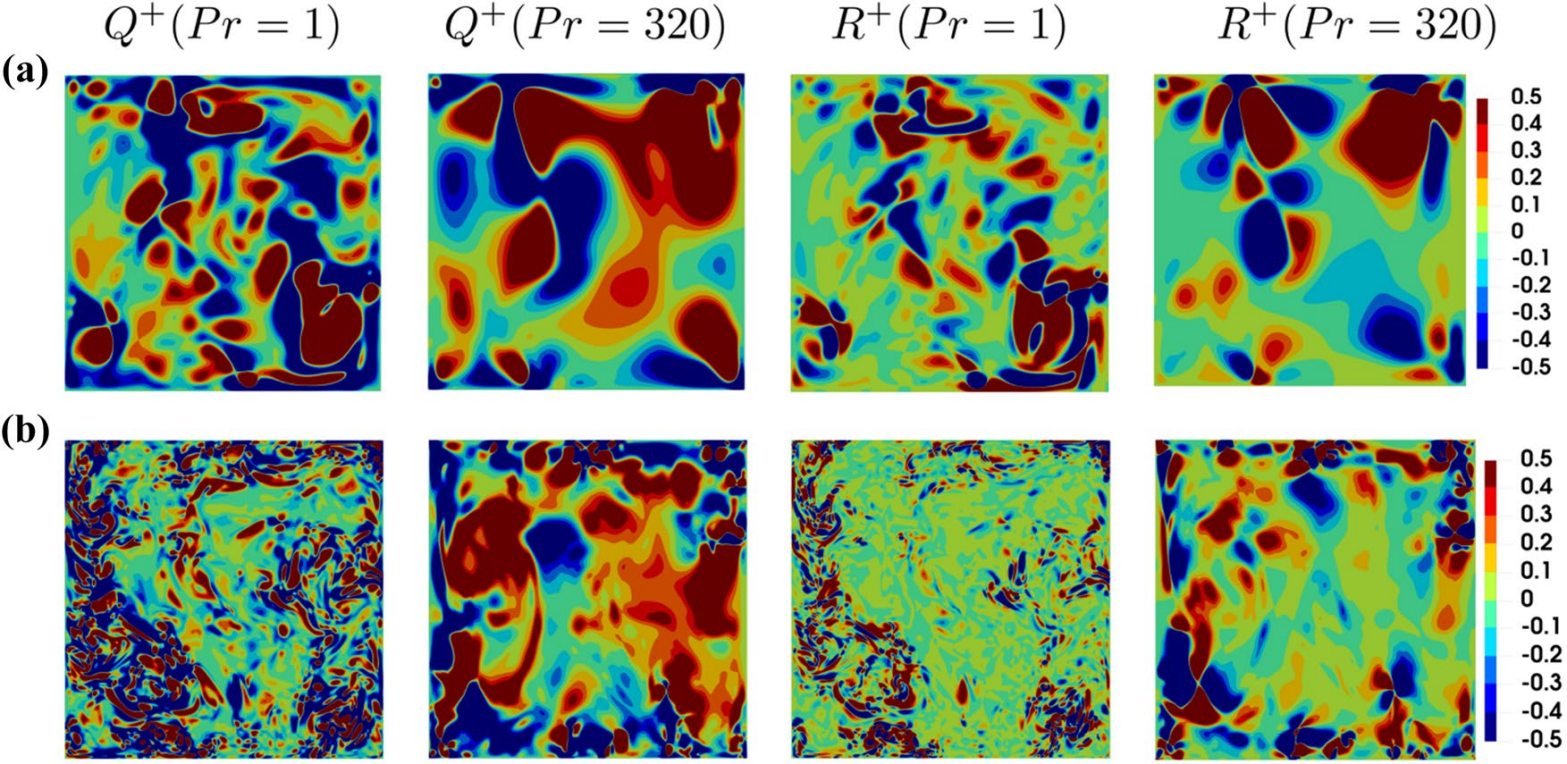
Instantaneous distributions of $Q^{+}$ and $R^{+}$ for different *Pr* values at **(a)** $Ra=10^7$ and **(b)** $Ra=10^9$ in the central $x_1-x_2$ plane.

The distributions of *Q* and *R* normalised by their standard deviations $Q^+={Q}/{\sigma }\left( Q\right)$ and $R^+={R}/{\sigma }\left( R\right)$ in the central $x_1-x_2$ plane for $Ra={10}^7$ and ${10}^9$ are shown for both $Pr=1$ and 320 in Fig. 3. For incompressible fluids *Q* is given by: $0.5\left( -S_{ij}S_{ij}+W_{ij}W_{ij}\right)$ and thus a positive (negative) value of *Q* is indicative of the vorticity-dominated (strain rate-dominated) regions. It can be seen from Fig. 3 (top row) that the vorticity-dominated regions (i.e. $Q^{+}>0$) are predominantly obtained in the central core of the enclosure away from the wall. By contrast, the strain rate dominated (i.e. $Q^{+}<0$) regions are predominantly concentrated in the vicinity of the wall within the boundary layer.

The differences in the $Q^+$ distributions between the near-wall region and away from the wall regions can be explained by the large scale or plume dominated mean flow as it is shown in Fig. 2. The flow in the bulk region is driven by the buoyant force, whereas large velocity gradients can be found predominantly in the boundary layer region (i.e. strain rate-dominated areas). Therefore, this difference between bulk and boundary layer region changes depending on the dominant physical mechanisms. The dissipation rate of kinetic energy $E =2\nu S_{ij}S_{ij}$ can be expressed as: $E =\nu (-4Q_{S})$ with $Q_{S}=-S_{ij}S_{ij}/2$ which suggests that $Q_{S}=0.25(- E)/\nu$ assumes large negative values in the regions where the dissipation rate of kinetic energy remains large within the boundary layer^25^. The dissipation rate of kinetic energy weakens with increasing wall normal distance, and thus the magnitude of negative $Q_{S}$ values decreases with increasing wall normal distance, which in turn increases the propensity of the positive semi-definite values of $Q_{W}=W_{ij}W_{ij}/2$ (i.e. $Q_{W}\ge 0$) to overcome negative values of $Q_{S}$ to yield positive values of $Q=Q_{S}+Q_{W}$ away from the wall (i.e. in the bulk flow region in the middle of the enclosure).

**Figure 4:**
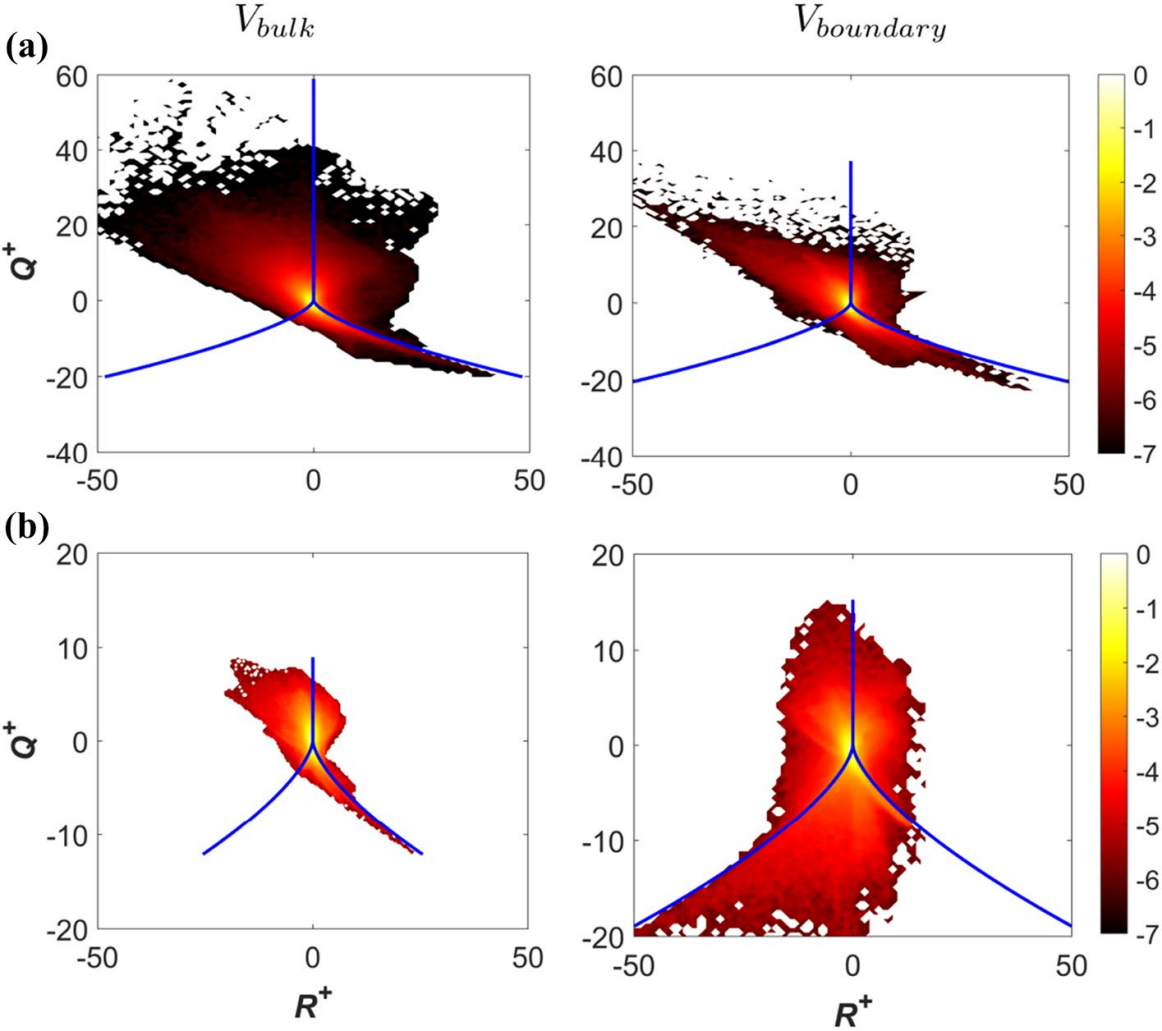
Contours of joint probability density functions of $Q^{+}$ and $R^{+}$ on a logarithmic scale in the bulk region (1st column) and at the heated and cooled boundaries (2nd column) for $Ra={10}^8$ in the case of **(a)** $Pr=1$, **(b)** $Pr=320$.

For $P=0$, the third invariant *R* takes the form: $R=(-S_{ij}S_{jk}S_{ki}-3W_{ij}W_{jk}S_{ki})/3=(-S_{ij}S_{jk}S_{ki})/3-\omega _i\omega _jS_{ij}/4$ where $\omega _i$ is the $i{\rm^{th}}$ component of vorticity. It is important to note that ($-S_{ij}S_{jk}S_{ki}$) contributes to dissipation rate $E=({2\mu }\,/{\rho })S_{ij}S_{ij}=\tau _{ij}(\partial u_i/\partial x_j)/\rho$ generation (with $\tau _{ij}$ being the component of the viscous stress tensor), whereas ($\omega _i\omega _jS_{ij})$ contributes to the production rate of enstrophy (i.e. $\Omega =\omega _i\omega _i/2$). Therefore, the sign of the $R$ indicates the competition and relative strengths of the enstrophy production rate and the dissipation rate generation^16^. A comparison between $Q^{+}$ and $R^{+}$ fields in Fig. 3 reveals that in particular large negative values of $Q^{+}$ are mostly associated with large positive values of $R^{+}$ in the bulk region of the enclosure for both $Pr=1$ and 320 and this is particularly prominent for $Pr=1$. However, the near-wall behaviour is different for different Prandtl numbers. In order to demonstrate this behaviour, the contours of joint probability density functions of $Q^{+}$ and $R^{+}$ in the bulk region (defined as $V_{bulk}=\left\{ \left( x_1,x_2,x_3\right) |\ 0.1\le {x_1}/{L}\le 0.9\ \, \& \,\ 0.1\le {x_2}/{L}\le 0.9\ \, \& \,\ 0.1\le {x_3}/{L}\le 0.9\right\}$) and at the heated and cooled boundaries (i.e. in the volumes $V_{boundary}=\left\{ (x_1,x_2,x_3)\,|\,\, 0.1\le {x_1}/{L}\le 0.9 \,\, \& \,\, 0.1\le {x_3}/{L}\le 0.9 \,\, \& \,\, ({x_2}/{L}\le 0.1 \,\,\mathrm{or}\,\, {x_2}/{L}\ge 0.9)\right\}$) are exemplarily shown for $Ra={10}^8$ in Fig. 4 in the case of both $Pr=1$ and 320. Although Fig. 4 shows the expected and well-known teardrop shape suggesting predominance of S4 and S2 (and to some extent S1) topologies both in the bulk region away from the walls and at the hot and cold boundaries for $Pr=1$, the conventional teardrop shape is obtained only in the bulk region for $Pr=320$ and the lower tail of the joint PDF flips from an unstable node-saddle-saddle S2 topology towards a stable node-saddle-saddle topology S3 at the hot and cold walls. These differences in joint PDFs between *Q* and *R* are expected to have implications on the distribution of the flow topologies within the enclosure.

**Figure 5:**
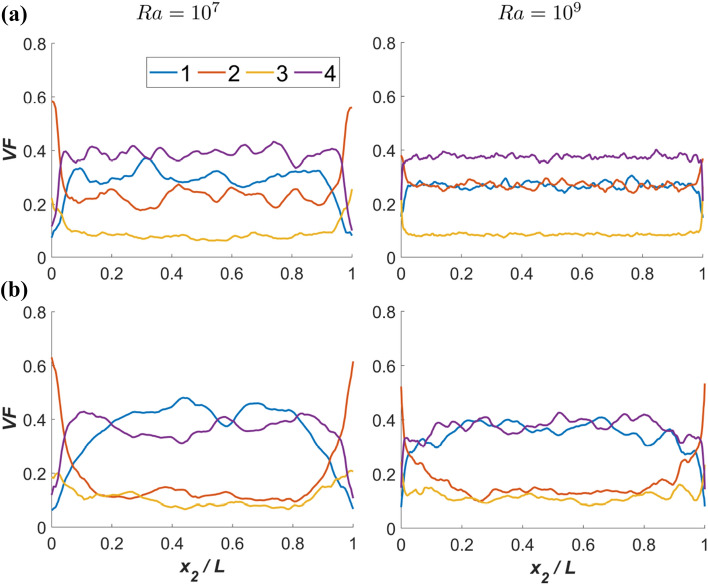
Variations of the volume fraction *VF* of flow topologies averaged in $x_1-x_3$ planes in the vertical direction (i.e. $x_2$-direction) are shown for $Ra={10}^7$ (1st column) and ${10}^9$ (2nd column) in the case of **(a)** $Pr=1$ and **(b)** 320.

The variations of the volume fraction *VF* of flow topologies averaged in $x_1-x_3$ planes in the vertical direction (i.e. $x_2$-direction) are shown in Fig. 5 for $Ra={10}^7$ and $10^9$ in the case of both $Pr=1$ and 320. It can be seen from Fig. 5 that $Pr=320$ (and to a lesser extent for $Pr=1$), the volume fraction of S1 and S4 topologies is about 40% in the bulk region, whereas the volume fraction of S2 and S3 is about 10% each within the bulk region of the domain regardless of *Ra* values. However, it is worth mentioning that the sum of both unstable and both stable topologies always seems to be very close to 50% for all values of *Pr* and *Ra*. This behaviour is consistent with the theoretical estimates by Hasslberger et al.^26^ assuming a symmetric population in $Q-R$ space. Close to the boundaries the volume fractions of nodal topologies (S2, S3) increase, whereas the volume fractions of focal topologies (S1,S4) decrease.

**Figure 6:**
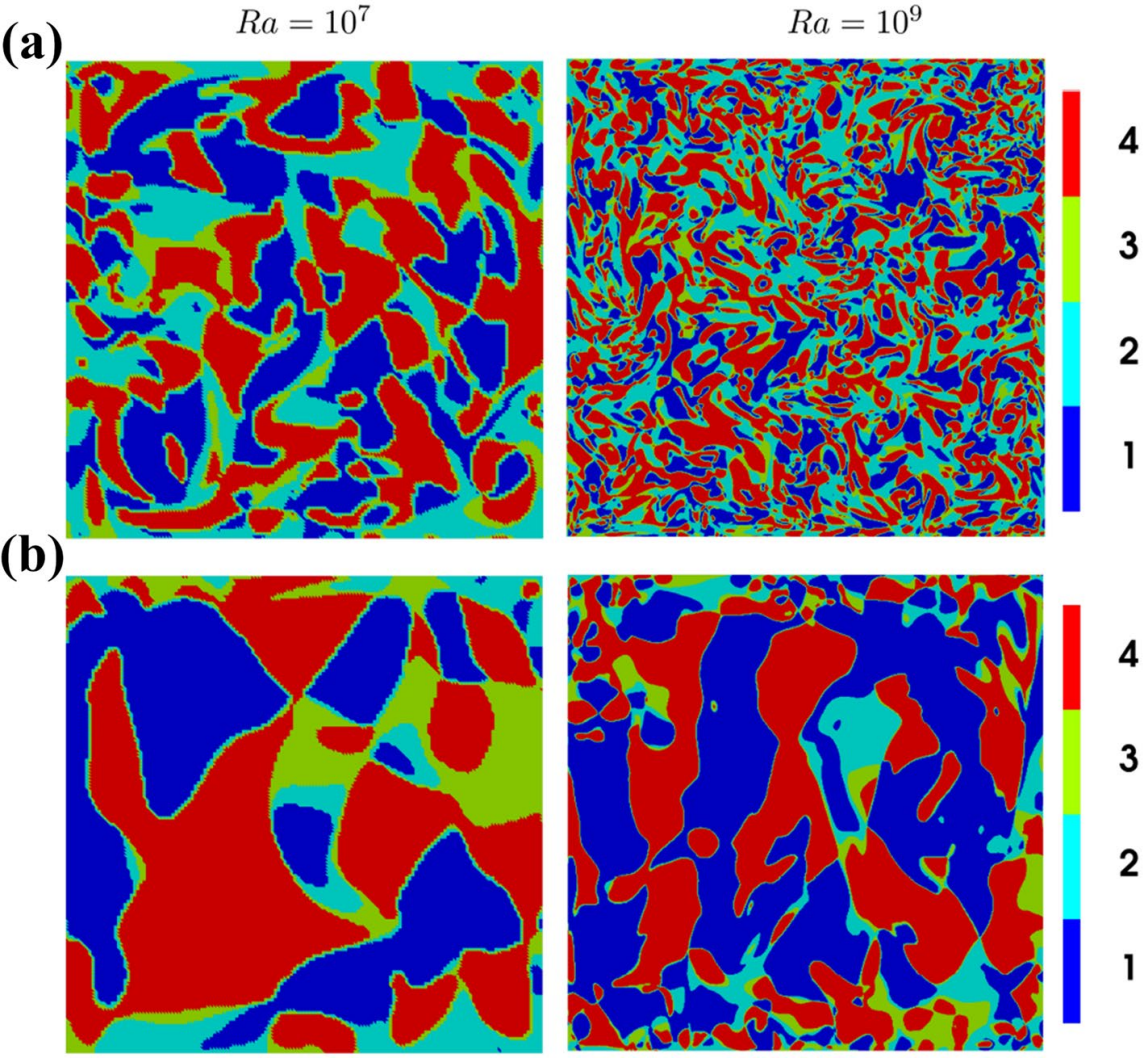
Instantaneous distrubitions of flow topologies in the central $x_1-x_2$ plane for $Ra={10}^7$ and ${10}^9$ at **(a)** $Pr=1$ and **(b)** 320.

Although results are shown only for $Ra={10}^8$ in Fig. 4, the distributions of $Q-R$ joint PDFs are qualitatively similar for the range of Rayleigh numbers considered in this study ($10^7 \le Ra\le 10^9$). This can be substantiated from the distributions of flow topologies in the central $x_1-x_2$ plane for $Ra={10}^7$ and ${10}^9$ at $Pr=1$ and 320, which are shown in Fig. 6. It can be seen from Fig. 6 that the focal topologies S1 and S4 are predominantly obtained in the bulk region at the interior of the domain, whereas the nodal topologies S2 and S3 are dominant in the vicinity of the hot and cold walls. This behaviour does not change with the variation in Rayleigh number within the $Ra={10}^7-{10}^9$ range considered here. However, the topology distribution in Fig. 6 suggests that the small-scale structures become more frequent for larger values of Rayleigh number and smaller values of Prandtl number which implies an increasing Grashof number.

**Figure 7:**
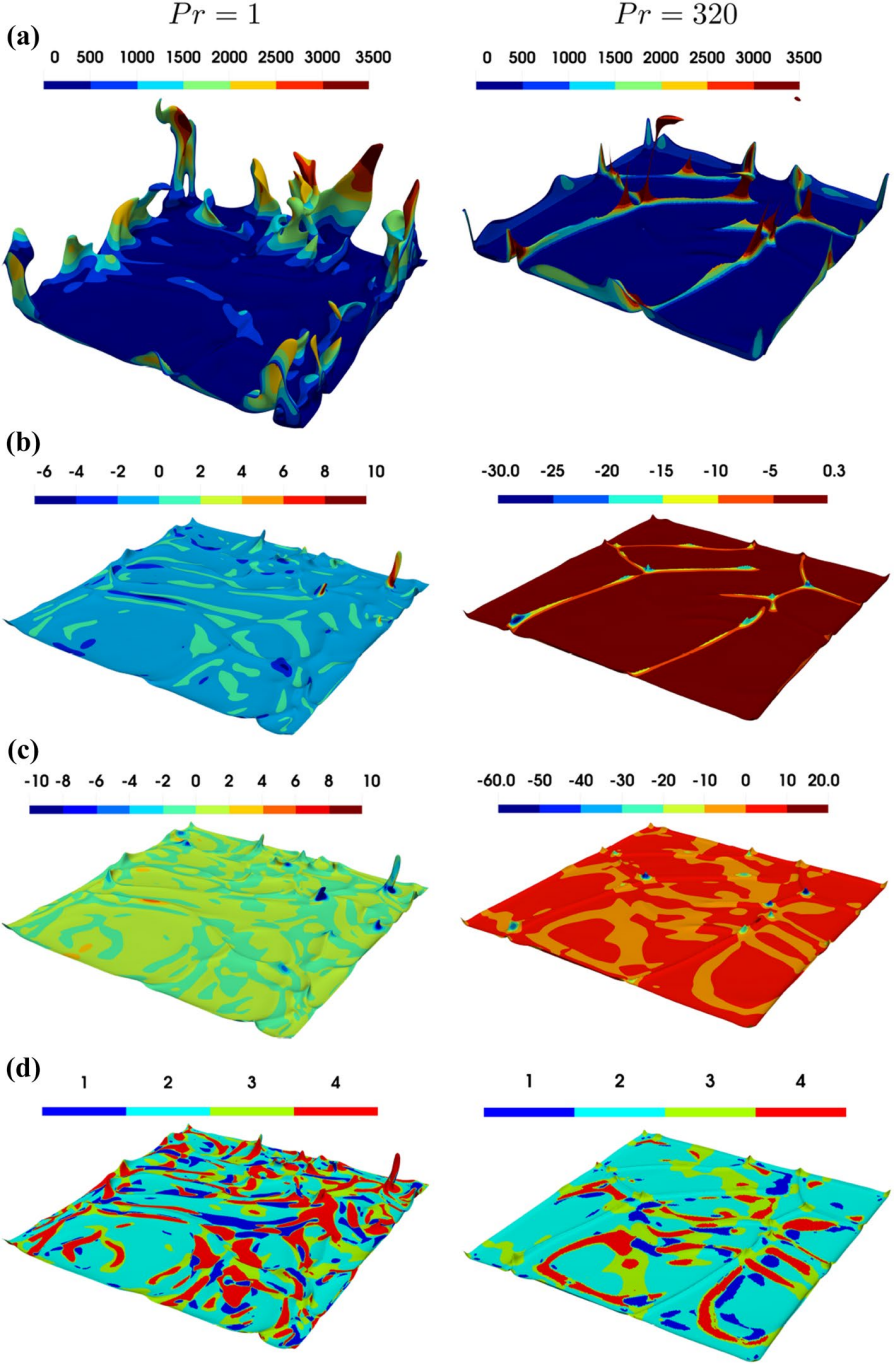
**(a)** Iso-surface of non-dimensional temperature $\theta =0.65$ coloured by non-dimensional vertical velocity *V* for $Ra={10}^8$ in the case of $Pr=1$ (1st column) and $Pr=320$ (2nd column). Distributions of **(b)** $Q^+$ (second row), **(c)** $R^+$ (third row) and **(d)** flow topologies (fourth row), on the $\theta =0.85$ iso-surface for $Ra={10}^8$ in the case of $Pr=1$ (1st column) and $Pr=320$ (2nd column).

In order to analyse the origin of the flipping of the tail of the joint PDF between *Q* and *R* towards the S3 quadrant, the region close to the bottom heated wall at $x_2=0$ is investigated further. The iso-surfaces of non-dimensional temperature $\theta =0.65$ (for better visibility of the structures $\theta =0.65$ is used in Fig. 7a) coloured by non-dimensional vertical velocity *V* for $Ra={10}^7,{10}^8$ and $Pr=1,\,\ 320$ are shown in Fig. 7 (first row) together with the corresponding distributions of  $Q^+$ and $R^+$ and flow topologies on the $\theta =0.85$ isosurface (second to fourth row). The ridge like structures for $Pr=320$ correspond to plume regions with large wall normal velocities directed away from the wall. The peaks where two ridges meet each other will subsequently be called junction points and a comparison with the flow topologies reveals that the junctions can be associated with topology S3. However, it can be seen that the S3 topology can also be observed in the valleys in between the ridges. In order to identify the origin of the reverse tail of the joint PDF between $Q^+$ and $R^+$ it is instructive to identify the regions with large negative values of $Q^+$ and $R^+$ and these quantities are mapped onto the iso-surfaces of non-dimensional temperature $\theta =0.85$ as well in Fig. 7. It becomes obvious from Fig. 7 that for $Pr=320$ there is a one-to-one relation between the junctions and the locations responsible for exhibiting S3 topology which is observed for the lower-left tail of the joint PDF between $Q^+$ and $R^+$ (i.e. for large negative $Q^+$ and $R^+$).

**Figure 8:**
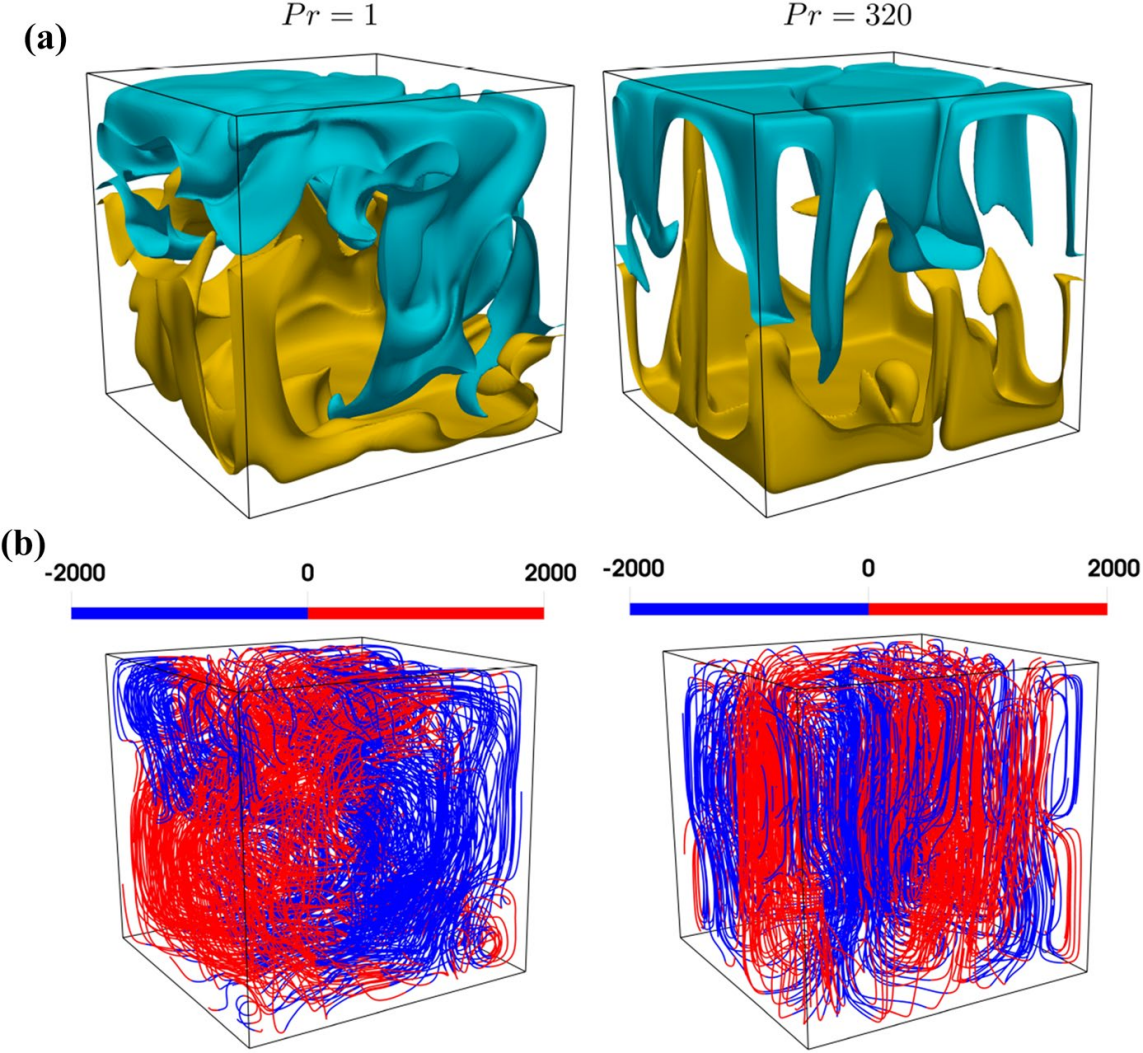
**(a)** Non-dimensional temperature iso-surfaces $\theta =0.4$, $\theta =0.6$ shown with blue and yellow colour, respectively. **(b)** Path lines coloured by non-dimensional vertical velocity magnitude (i.e. $V=u_{2}L/\alpha$ ) for different *Pr* at $Ra={10}^7$.

Figure 2 reveals that isolated plumes drive the convection process in the $Pr=320$ case, whereas frequent roll ups in the $Pr=1$ case are indicative of a large-scale circulation. This is further illustrated in Fig. 8 showing the variation of non-dimensional temperature iso-surfaces $\theta =0.4$ and  $\theta =0.6$ together with path lines coloured by non-dimensional vertical velocity magnitude for different *Pr*, exemplarily at $Ra={10}^7$. This behaviour is consistent with previous findings by van der Poel et al.^21^ and Verzicco and Camussi^27^. Figure 7 further reveals that it is rare to obtain simultaneous occurrences of large negative values of $Q^+$ and $R^+$ in the near wall region for $Pr=1$, and accordingly the S3 topology is rare (and S2 is dominant, cf. *VF* profiles in Fig. 5) in the vicinity of the wall in particular in connection with large negative values of $Q^+$ and $R^+$. An extraction of local flow structures with large negative values of $Q^+$ and $R^+$ (not shown here) reveals that that the local flow structures responsible for the S3 tail of the joint PDF can indeed only be found in the vicinity to the active walls for $Pr=320$ and such structures are entirely absent for $Pr=1$. A comparison between the schematic flow diagram in Figs. 1 and 7 reveals that the plumes in the $Pr=320$ case are representative of the S3 topology and thus the occurrence of S3 with large negative values of $Q^+$ and $R^+$ are more likely in this value of *Pr*, which is also reflected in the flipping of the tail of the joint PDF between $Q^+$ and $R^+$ to the quadrant of S3 topology. As the convection is driven by large-scale circulation in the $Pr=1$ case, the occurrences of S3 topology with large negative values of $Q^+$ and $R^+$ are either rare or absent.

## Summary

The statistical behaviours of the invariants of the velocity gradient tensor and flow topologies in turbulent Rayleigh–Bénard convection of Newtonian fluids in cubic enclosures have been investigated using three-dimensional Direct Numerical Simulations (DNS) for a range of different Rayleigh (i.e. $Ra={10}^7-{10}^9$) and Prandtl numbers (i.e. $Pr=1$ and 320). It has been found that the convection in the case of large values of *Pr* is plume-dominated, whereas a large-scale circulation in the enclosure has been found for $Pr=1$. The focal topologies S1 and S4 have been found to be the two dominant topologies in the bulk region, whereas the probabilities of obtaining nodal topologies S2 and S3 increase in the vicinity of the active hot and cold walls. The proportion of different flow topologies in the bulk region has been found to be consistent with a previous analytical study^27^ assuming a symmetric population in the two-dimensional phase space based on second and third invariants of the velocity gradient tensor (i.e. *Q* and *R*). The classical teardrop shape of the joint PDF between *Q* and *R* has been observed away from active walls, but this behavior changes close to the heated and cooled walls where the joint PDF depicts a shape mirrored at the vertical *Q*-axis for large values of Prandtl number (e.g. $Pr=320$). It has been demonstrated that junctions, at the edges of convection cells arising from plume-dominated convection, are responsible for this behavior for $Pr=320$. This behaviour is not present in the $Pr=1$ case due to the convection driven by a large-scale circulation. The differences between flow topology distributions in Rayleigh–Bénard convection in response to *Pr* suggest that turbulence modelling of natural convection possibly should explicitly account for Prandtl number effects. Therefore, the modelling methodologies for simulations of turbulent natural convection of gaseous fluids may not be equally well-suited for natural convection of liquids with large values of Prandtl number.

## Methods

The governing equations of mass, momentum and energy conservation equations have been solved in a finite-volume framework using an open-source CFD package OpenFOAM. For these computations pressure–velocity coupling has been addressed by the use of the PIMPLE algorithm. The convective and diffusive fluxes are evaluated using second-order central difference schemes. The temporal advancement has been carried out using the second-order Crank-Nicolson scheme in conjunction with adaptive time-stepping for the sake of computational economy. It has been ensured that the Courant number is always below unity so that the simulations have enough temporal resolution. Ideally, the grid size $\Delta x$ for DNS of Rayleigh–Bénard convection should satisfy $\Delta x < min(\langle \eta _{K}\rangle ,\langle \eta _{B}\rangle )$ where $\langle \eta _{K} \rangle$ is the Kolmogorov length scale and $\langle \eta _{B}\rangle$ is the Batchelor length scale. These length scales are defined as:

6 $$\begin{aligned} \langle \eta _{K} \rangle = \nu ^{3/4}/{\langle \epsilon \rangle }^{1/4}; \,\langle \eta _{B} \rangle =\langle \eta _{K} \rangle /\sqrt{Pr} \end{aligned}$$

where $\langle \epsilon \rangle =\langle 2\nu \,{S_{ij}^\prime } \, {S_{ij}^\prime } \rangle$ is the mean kinetic energy dissipation rate with the angled bracket indicating a volume–time averaging technique. Here, $S_{ij}^\prime$ is the strain rate component based on the fluctuating velocity field and $\nu$ is the kinematic viscosity. The mean Kolmogorov scale $\langle \eta _{K} \rangle$ is the smallest length scale of turbulence when $\nu \le \alpha$ (i.e. $Pr\le 1$). In case of $\nu >\alpha$ (i.e. $Pr>1$), the smallest length scale is determined by the dissipation rate ${\langle \epsilon _{T} \rangle }=\langle \alpha \, {(\nabla T^ \prime )}^2 \rangle$ of scalar variance, and is represented by the Batchelor scale $\langle \eta _{B} \rangle$^4^. Criteria proposed by Grötzbach^28^ and Shishkina et al.^29^ have been used for determining the initial non-dimensional mesh size ($\Delta x/L$), along with the corresponding ${\overline{Nu}}$ correlations suggested by Refs.^23,30^.

Additionally, Grötzbach^28^ suggested that at least three grid points should be placed in the viscous boundary layers to estimate the realistic Nusselt number. Verzicco and Camussi^31^ and Stevens et al.^32^ found that the number of nodes that should be placed in the thermal and hydrodynamic boundary layers increases with increasing *Gr*. Based on this, the $Pr=1$ cases are more critical in terms of grid resolution than the $Pr=320$ cases, because the values of *Gr* are much higher for $Pr=1$ cases than the one in the $Pr=320$ cases for the same set of *Ra* (i.e. $Gr=Ra/Pr$). Lam et al.^33^ proposed a correlation for the normalised viscous layer thickness as $\delta /L=0.65\ {Gr}^{-0.16} {Pr}^{0.08}=0.65{Ra}^{-0.16} {Pr}^{0.24}$ based on experimental analysis of turbulent Rayleigh–Bènard convection of Newtonian fluids in a single convection cell of unity aspect ratio for ${10}^6\le \ Ra\le {10}^{11}$ and $6\le \ Pr\le {10}^3$. Using this normalised viscous layer thickness correlation^33^ more than 15 grid points have been placed in the $Pr=1$ cases for all the values of *Ra* (${10}^7-{10}^9$) considered in the current analyses.

Finally, it has been ensured a-posteriori that the chosen grid resolution is sufficient by ensuring $y^+$ and $y^+\sqrt{Pr}$ (where $y^+={\rho u}_\tau y/\mu$ with $u_\tau =\sqrt{\tau _w/\rho }$, $\tau _w$ and *y* being the friction velocity, wall shear stress magnitude and wall normal distance of the wall adjacent grid point, respectively) remains smaller than unity. For the present analysis, Cartesian grids of ${250}^3,\ {460}^3$ and ${700}^3$ (${150}^3,\ {230}^3$ and ${490}^3$) have been used for $Pr=1$ ($Pr=320$) simulations of $Ra={10}^7$, $Ra={10}^8$ and $Ra={10}^9$, respectively.
